# How to … Build a Peer Reviewer Community in Health Professions Education

**DOI:** 10.1111/tct.70009

**Published:** 2024-12-25

**Authors:** Jun Jie Lim, Laura Cheetham, Christopher J. Graham, Agata Anna Dunsmore, Aileen Barrett

**Affiliations:** ^1^ Division of Clinical Medicine, School of Medicine and Population Health University of Sheffield Sheffield UK; ^2^ Aneurin Bevan University Health Board Newport UK; ^3^ Department of Education, Training and Assessment Royal College of Physicians of Edinburgh Edinburgh UK; ^4^ Division of Clinical and Surgical Sciences University of Edinburgh Edinburgh UK; ^5^ Irish College of GPs Dublin Ireland

**Keywords:** community of practice, early‐career scholars, health professions education, mentorship, peer review, peer reviewer training

## Abstract

Peer review is the cornerstone of academic publishing that upholds the quality and integrity of scholarly work. However, there is an ever‐growing struggle to recruit peer reviewers, termed the ‘peer review crisis’, driven by a shrinking academic workforce and increased workload. Additionally, there is a notable lack of standardised training for peer reviewers which poses a challenge in maintaining high‐quality reviews. In this ‘How to …’ paper, we demonstrate the feasibility and benefits of establishing a community of practice (CoP) aimed at fostering professional development among multiprofessional early‐career scholars in health professions education. A CoP is structured around three core components: *domain*, *community* and *practice*. This community's *domain* focuses on recruiting scholars with a shared interest in health professions education, research and peer review. The *community's* component promotes inclusive and regular interactions through synchronous meetings and asynchronous communication, encouraging engagement, mutual learning and collaboration among diverse participants. *Practice* is cultivated through peer‐led teaching sessions and the use of digital platforms, enabling participants to build peer review competencies collaboratively. Based on our experience as participants in the Association for the Studies of Medical Education (ASME) and *The Clinical Teacher* (TCT) awarded programme of professional development in educational research and peer review, we propose that this model may help other institutional health professions education groups and journals adopt similar practices. Providing early‐career scholars with opportunities to develop academic skills will not only help create a sustainable, high‐quality pool of peer reviewers but also cultivate a more inclusive and skilled scholarly community.

## 1 Introduction

Peer review is a cornerstone of academic publishing, upholding academic integrity, rigour and credibility [1]. However, the process is under increasing strain, with most journals facing challenges in recruiting reviewers—a ‘peer review crisis’. This crisis is driven by factors such as an overburdened academic workforce and a rising number of competing journals [2]. A global survey found that 75% of journal editors face challenges in recruiting peer reviewers [3]. Compounding this issue is the lack of standardised training or accreditation for peer reviewers, who are often called upon out of goodwill, and leaving the development of peer review skills to inconsistent methods like workshops or informal guidance [4].

Besides logistical challenges, the ethos of peer review is also evolving. Reviewers should not only uphold academic integrity but also provide constructive feedback that enhances the quality of scholarly work. However, this developmental aspect is often overlooked. Health professions education (HPE) journals, in particular, face added difficulties with a smaller pool of reviewers and limited access to scholars from low‐ and middle‐income countries [4].

Although current narratives have focused on creating solutions to the peer review crisis, including offering financial and professional incentives to reviewers, these do not ensure long‐term sustainability [5]. Building professional capacity by engaging more early‐career scholars as reviewers could be a promising approach to alleviating the current shortage and enriching the peer review process by welcoming diverse perspectives and fostering a more inclusive scholarly dialogue [6]. However, there is limited guidance for early‐career scholars to gain the necessary experience and build confidence to engage in the peer review process meaningfully.

In this ‘How to …’ paper, we demonstrate the feasibility and benefits of establishing a CoP for multiprofessional early‐career peer reviewers, fostering professional belonging and skill development. Our insights are drawn from existing literature, our experiences as the Association for the Studies of Medical Education (ASME) and *The Clinical Teacher* (TCT) New Voices in HPE awardees and apprentice peer reviewers, as well as from the perspective of the programme's conceptor and former editor‐in‐chief of TCT.

### 1.1 Context

ASME and TCT launched the ‘New Voices’ award programme in 2021. Designed to support early‐career HPE scholars, this initiative aims to develop peer review skills while amplifying voices that have historically lacked access to such opportunities due to geographic or other barriers [7]. This 9‐month, fully virtual programme utilises a community of practice (CoP) model [8] to build peer review expertise, confidence, a sense of belonging and professional growth among a diverse, multidisciplinary group of early‐career scholars from around the world. The template for intervention description and replication (TIDieR) checklist [9] is used as a guide for how others may replicate this programme (Table 1).

**TABLE 1:** Summary of the ‘New Voices’ programme using an adapted TIDieR checklist [8].

Item	Description
Name	*The Clinical Teacher* ‘New Voices in Health Professions Education’ Award
Why	To equip early‐career HPE scholars with essential skills and knowledge for effective peer review. This addresses the shortage and enhances the quality of peer reviews in HPE journals. The programme is underpinned by CoP theory.
What	A structured, progressive online programme of mentored peer reviewing, facilitated by the journal's editorial team and awardee‐peers. The programme includes monthly online tutorials on topics related to HPE scholarship, guided peer reviews and near‐peer teaching sessions. Participants receive free access to ASME membership benefits, which include resources for peer review and access to ASME journals (*Medical Education* and *The Clinical Teacher*).
Who provided	Journal editors and deputy editors from *The Clinical Teacher* scaffold tutorials, lead sessions and provide mentorship. They share knowledge on how to approach peer review and ensure quality in the process. Awardees deliver peer teaching and guests such as a journal inclusivity lead are invited to participate during select meetings.
How	Virtually via a combination of whole group meetings using videoconferencing platforms for tutorials, discussions and guided peer review sessions. Whole group and subgroup asynchronous discussions via email, closed social media platforms and shared working documents.
Where	Videoconferencing platforms like Zoom Workplace and Microsoft Teams enhance accessibility for a wide range of participants. Additionally, closed social media platforms, such as WhatsApp, and collaborative document‐sharing platforms, like Google Drive and Google Workspace, have been adopted as part of the evolving CoP.
When and how much	The programme runs over a nine‐month period with monthly video meetings lasting 1 hour each. Each session is structured as follows: the first 30 min dedicated to peer‐led teaching on a pre‐allocated topic related to HPE scholarship, followed by 30 min of subgroup discussions and reflection on prepared critiques of an academic paper (flipped classroom approach). Participants are expected to commit to ongoing peer reviewing beyond programme completion.
Tailoring	There was no specific ‘tailoring’; all awardees are provided with the same opportunities.
Modification	No major modifications were made to the programme during its delivery. Minor changes included increasing the number of annual awardees and a shift in co‐leads during the 2023–2024 award cycle due to editor succession.
How well (planned) and how well (delivered)	The programme was delivered as planned with sustained engagement and attendance throughout.

### 1.2 Theoretical lens

A CoP is defined as groups with shared interests that interact to deepen expertise [10]. In our context, the *domain* refers to our shared subject of interest—peer review. As early‐career scholars gain proficiency in peer reviewing, they become legitimate members of the CoP. The *community* forms the social structure that supports the exchange of ideas, critiques and shares insights among trainees and editors. This framework encourages collaboration, fosters mutual respect and enables the constructive dialogue necessary to develop skills in peer review. The *practice* consists of the methods and tools used to evaluate and improve scholarly work and the specific knowledge and skills that the peer review community collectively develops.

## 2 How to … Establish a ‘Domain’ in a Peer Review CoP

### 2.1 Recruit Early‐Career Scholars Through Diverse Communication Channels

Creating the *domain* begins with gathering early‐career scholars from various healthcare professions who show enthusiasm to contribute to the academic community. A standardised recruitment process ensures that CoP members are selected transparently and fairly, asking applicants to submit a summary document outlining their interest in HPE and an ‘elevator pitch’ video giving an introduction and outlining career goals. Each application, along with a two‐page curriculum vitae, was evaluated by the journal editors. Initial recruitment faced challenges, leading outreach to be extended via multiple platforms and social media to ensure broader inclusion.

### 2.2 Set Mutual Expectations and Learning Objectives

Understanding each other's objectives is essential to establishing the *domain* by setting clear mutual expectations and providing structure within the CoP. This could include regular attendance at meetings, peer‐led teaching of academic concepts and preparation of a critique of academic writing for peer discussions and reflection. This approach is grounded in social constructivism [11], with structured commitments fostering relationships where learners co‐construct knowledge through each other's experiences and insights. Upon completion, awardees are encouraged to continue contributing to the field by peer reviewing for TCT.

The CoP facilitates networking opportunities that encourage participants to develop and submit articles to TCT. Furthermore, awardees may act as peer reviewers for other CoP members' work. This familiarises early‐career scholars with how to develop scholarly ideas, collaborate and disseminate research. Participants of the CoP thus become accustomed to journal procedures and processes. Community members who may face challenges in contributing due to clinical or personal commitments may still participate through asynchronous activities. This approach helps create a positive and inclusive environment.

### 2.3 Editors as Leaders and Facilitators

The *domain* is further reinforced through the involvement of journal editors who act as both leaders and facilitators of the CoP. Editors guide the learning process by organising sessions, selecting themes for peer‐led teaching and choosing papers for appraisal. The journal editors are experienced academics and researchers, who enable participants to work at the limit of their expertise, within Vygotsky's zone of proximal development, to develop skills that otherwise may be more difficult to gain individually [12].

## 3 How to … Foster a ‘Community’ in a Peer Review CoP

### 3.1 Provide Structure, With Room for Adaptation

Regular meetings are crucial for fostering engagement and relationship building within the CoP. Conversations continue before and after meetings through digital, asynchronous means. This allows participants space and time to reflect and develop ideas away from formal tutorials. This approach embodies the principles of social constructivism and sociocultural learning theory, which emphasise the importance of discussion in knowledge acquisition [13]. The international, multiprofessional cohort of awardees means that there is a diverse range of perspectives, which adds depth to discussion and richness in learning. Meeting schedules should be collaboratively decided upon to accommodate participants' diverse needs. The design of these interactions should also be crafted in partnership with CoP members to promote inclusivity and sustained involvement.

While we were fortunate to reach a wider audience in our later efforts, this also resulted in challenges in arranging synchronous meetings that were consistently accessible to all awardees. When some participants had to join at unsociable hours due to time zone differences, we explored alternatives to better support the diverse needs of the group. One option considered was repeating the meeting twice at different times while another suggestion was to find a middle ground by scheduling meetings at a more convenient time for this minority. Due to logistical challenges, we were unable to adopt either approach, but we would recommend that others consider trying these strategies to accommodate diverse time zones more effectively.

### 3.2 Collaborative Learning in Subgroups

In addition to participating in the main CoP group, members are divided into smaller subgroups. Within subgroups, individuals interact synchronously and asynchronously through email, videoconferencing tools and messaging platforms. These subgroups also facilitate collaborative peer review sessions in a flipped classroom format, where members undertake peer reviews before the main meetings, scaffolded by questions provided by the journal editors. This approach supports self‐directed learning and a safe space where all participants feel able to speak up. Furthermore, these subgroups facilitate interprofessional learning by enabling participants to engage deeply with peers from various healthcare disciplines through an exchange of ideas, methods and diverse professional insights [14]. As a result, CoP members build stronger connections and develop sustained peer‐to‐peer learning relationships.

### 3.3 Integrate CoP Members Into the Wider HPE Community

Participants are integrated into the HPE community through ASME membership, funding to attend conferences and opportunities to chair sessions and present work. This fosters a sense of belonging, allowing them to expand their professional networks and deepen their involvement in HPE. Additionally, other early‐career scholars may be inspired to join the CoP, further expanding the community.

## 4 How to … Build a Shared ‘Practice’ Within a Peer Review CoP

### 4.1 Peer‐Led Teaching Sessions

The programme is enhanced by peer‐led tutorials that promote active learning. During these sessions, each subgroup delivers a peer teaching session based on editor‐selected topics relevant to peer review (Table 2). These topics are chosen to align with the needs and interests of the CoP. Presenters receive feedback, developing academic skills while fostering collaboration.

**TABLE 2:** Topic list for teaching sessions.

Session	Topic
1	Peer review—an overview
2	Quality in qualitative research and ethical issues for educational research
3	‘The problem, gap and hook’—academic writing in HPE research and scholarship
4	Theory, conceptual and theoretical frameworks in HPE research
5	Quality improvement in HPE
6	Equality, diversity and inclusivity in research, peer review and publishing
7	Best practice for peer reviewing

### 4.2 Effective use of Communication Platforms

**FIGURE 1:**
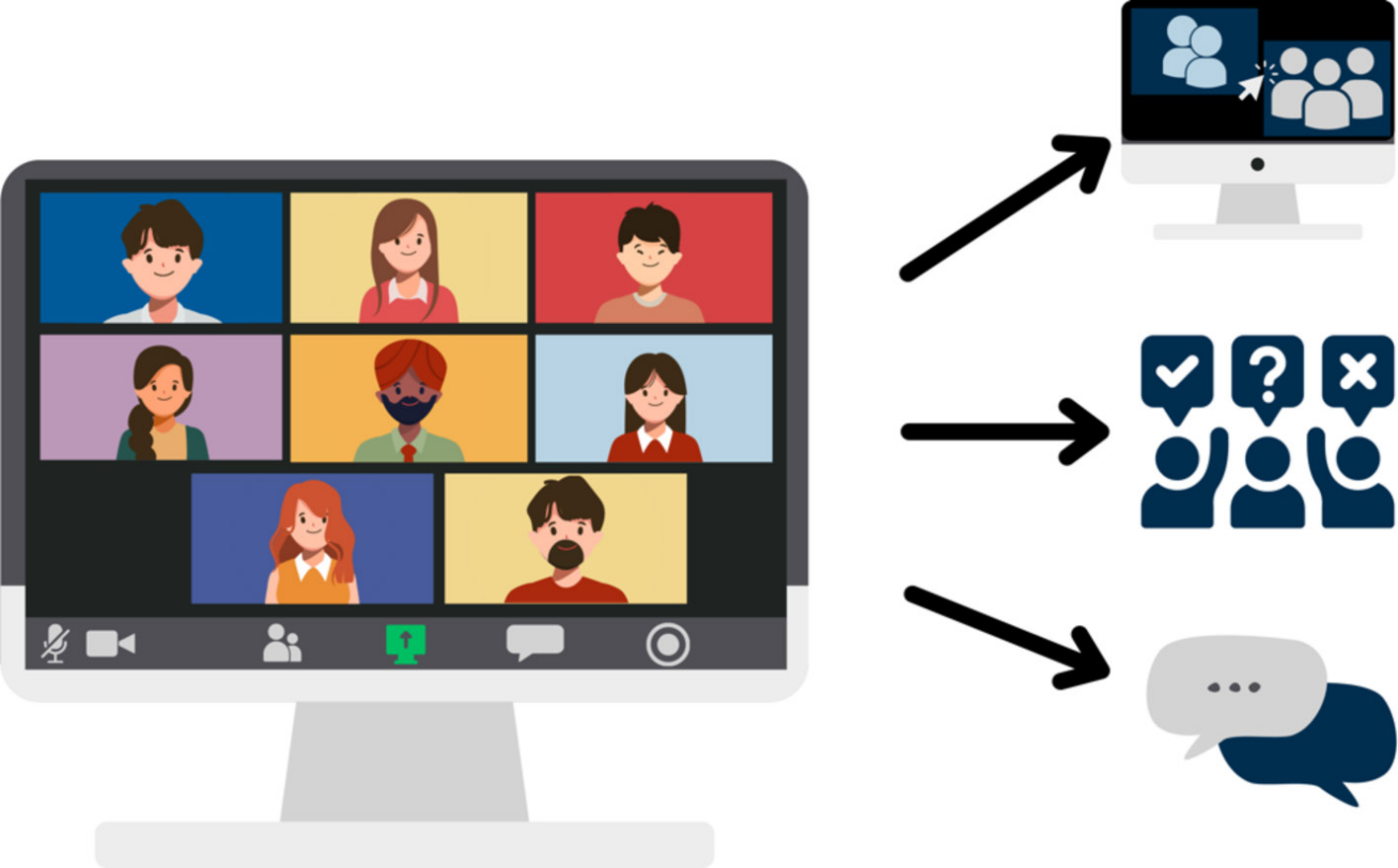
The features of videoconferencing platforms (breakout rooms, polls and chat functions). *Note*: Figure 1 was created by the author, J.L., using Canva Pro in accordance with Canva’s Pro Content Licence. All design elements and graphics were sourced through Canva’s Pro service, adhering to their licensing terms.

Choosing the optimal communication platform(s) for a CoP is essential to encourage participation as technical issues and poor user experience can hinder engagement. Various virtual platforms offer different features, such as breakout rooms for small group discussions, anonymous polls and chat functions to interact or share resources (Figure 1). The software should be free and ubiquitous for awardees and have features that support how meeting interaction is structured. More than one platform may be used; for example, additional closed social media platforms such as WhatsApp enable interaction between formal meetings to share knowledge and resources via various device types.

## 5 Promoting EDI Within the CoP

Equality, diversity and inclusion (EDI) principles are embedded throughout the programme to ensure diversity. Outreach includes promotion via social media and to low‐ and middle‐income countries. The application process is inclusive, allowing applicants to showcase their strengths through video and written submissions. Psychological safety is prioritised [15], with flat hierarchies and inclusive language fostering a constructive environment (outlined in Box 1). The programme is free, to eliminate financial barriers [16].

Box 1 **Suggestions to promote psychological safety within a peer review CoP.**

- Building familiarity on a first‐name basis.
- Creating and maintaining a flat hierarchy.
- Providing various means of participation (e.g., polls and chat).
- Editors encouraging participation through a variety of modes.
- Welcoming and valuing all opinions and contributions regardless of professional background.
- Editors expressing respect and high regard for learners.
- Maintaining an open‐door policy for awardees to contact editors.

## 6 Implications and Future Suggestions

Our virtual CoP enables early‐career scholars to build a network of scholarly relationships. This could provide a feasible, sustainable and inclusive route to reduce the current attrition of peer reviewers while enhancing the development of diversity within the early‐career scholar community. To further strengthen this approach, integrating a peer review CoP with alumni mentoring could create a sustainable, self‐perpetuating cycle of skill development and knowledge sharing. Newly trained peer reviewers can mentor novice scholars, fostering a spiral of near‐peer learning [17]. We recommend similar initiatives for other institutional health professions education groups, journals and publishers. The model could also be applied across HPE and higher education institutions. To conclude this ‘How to …’, we share personal reflections as participants in this CoP and offer recommendations for early‐career scholars to encourage engagement in similar initiatives (see Box 2).

Box 2 **Personal reflection and recommendations from CoP members.**

Personal reflection from authors

Jun Jie Lim—‘I joined the programme to improve my peer reviewing and critical appraisal skills. It has been incredible to learn from this community of early‐career scholars and friendly editors. Most importantly, we motivate each other in navigating the route of HPE scholarship which would have otherwise been lonely and tortuous.’

Laura Cheetham (Josie)—‘The New Voices Award programme has been a highlight of the past year for me. It is a gateway to joining a diverse, talented and enthusiastic community of early career scholars. Being part of the Award programme has enabled me to explore the peer review process at a level of detail and from a range of lenses rarely accessible elsewhere. This has prepared and further enthused me for future scholarly activities and stepping into peer review positions with greater confidence.’

Chris Graham—‘The “New Voices” award provides a unique opportunity to learn from a diverse and passionate community of early‐career scholars and the expert editorial team. Critical thinking and peer review are essential skills to develop, so I encourage you to take advantage of this supportive programme and apply.’

Agata Dunsmore—‘I am extremely grateful to be part of this new community. I had done two peer reviews prior to starting this award which I accepted reluctantly. I lacked confidence in the validity of my comments to the authors and unsure if it was me not understanding the paper. The past nine months have helped me develop my knowledge, skills and self‐belief but most of all, I have friends I can ask.’

## Author Contributions

**Jun Jie Lim:** writing – original draft, writing – review and editing, visualization. **Laura Cheetham (Josie):** writing – original draft, writing – review and editing, visualization. **Christopher J. Graham:** writing – review and editing, visualization, writing – original draft. **Agata Anna Dunsmore:** conceptualization, writing – review and editing, visualization. **Aileen Barrett:** writing – review and editing, supervision, visualization.

## Ethics Statement

Ethical approval was not required for this study.

## Conflicts of Interest

J.L., J.C., C.G. and A.D. are recipients of *The Clinical Teacher* ‘New Voices in Health Professions Education’ Award 2023. A.B. is the former editor‐in‐chief of *The Clinical Teacher* and co‐established *The Clinical Teacher* ‘New Voices in Health Professions Education’ Award programme.

## Data Availability

Data sharing is not applicable to this article as no datasets were generated or analysed during the current study.
